# Pneumonia and pneumothorax detection: A multi-factor evaluation of chest X-rays

**DOI:** 10.1371/journal.pone.0341060

**Published:** 2026-01-20

**Authors:** Yousef Saad Aldabayan

**Affiliations:** Department of Respiratory Care, College of Applied Medical Sciences, King Faisal University, Al-Ahsa, Saudi Arabia; Najran University College of Computer Science and Information Systems, SAUDI ARABIA

## Abstract

The research creates a Vision Transformer (ViT) diagnostic system which identifies pneumonia and pneumothorax from chest radiographs through analysis of the NIH ChestX-ray14 dataset. The research methodology solves medical imaging problems through three essential components which include (i) radiograph-specific augmentation for simulating authentic imaging conditions and (ii) multi-label imbalance handling through WeightedRandomSampler with class-specific weight application to stop all-normal predictions and (iii) optimization improvements that include CosineAnnealingWarmRestarts scheduling and sigmoid-based classification head optimization and disease-specific threshold optimization. The evaluation of model performance uses AUC and sensitivity and specificity and precision and F1-score because accuracy proves ineffective when dealing with severe class imbalances. The ViT models achieve 70−75% accuracy and 0.63–0.71 AUC values for both target conditions during non-leaking and noise-aware experiments because of the weak labels and restricted supervision in the ChestX-ray14 dataset. The system enhances its ability to detect rare conditions while providing better interpretability through Vision Transformer attention-based visualization of important radiological areas. The research demonstrates that ViT performance improves significantly through medical-focused data preparation methods and training approaches which demonstrate potential for radiology assistance in high-volume and resource-constrained environments.

## Introduction

The worldwide medical community faces two leading thoracic diseases which cause numerous deaths and illnesses because they need immediate proper diagnosis to receive proper treatment. The research investigates the challenge of accurately identifying pneumonia and pneumothorax through chest X-ray8 (CXR) images in settings with large patient numbers and limited resources. The disease causes pneumonia to develop inflammatory exudates and parenchymal consolidation which leads to widespread opacification that can result in respiratory failure when treatment is delayed [1,2]. The accumulation of air in the pleural cavity results in pneumothorax which causes lung collapse and severe respiratory problems and cardiovascular instability through mediastinal displacement thus requiring immediate diagnosis to stop fatal progression [3,4]. Misdiagnosis of either condition can therefore have profound clinical consequences. The tool serves as a widely used diagnostic tool because it provides fast results at affordable prices [5,6], the process of interpreting chest X-ray images contains specific challenges because it requires specialized skills that humans can only perform with difficulty. The evaluation process becomes difficult because of multiple small radiographic indicators and different patient placement methods and varying exposure levels and multiple overlapping medical conditions. The evaluation of radiologic images by experienced radiologists remains inconsistent because they disagree when interpreting unclear or poorly defined medical images [4,6–8]. The situation becomes worse in rural areas with limited resources because trained radiologists are scarce which leads to longer diagnostic times and higher workloads for medical staff [7,8]. The ongoing diagnostic challenges require dependable computer-aided diagnostic (CAD) systems which should assist clinicians through better interpretation accuracy and reduced workload and enhanced detection of missed diagnoses [9,10].

Medical image classification benefits from Deep learning methods which use Convolutional Neural Networks (CNNs) as their primary approach [11]. The application of CNN-based methods to CXR interpretation faces multiple built-in restrictions. The system fails to detect long-range spatial dependencies because it uses localized receptive fields which makes it impossible to identify diffuse pneumonia opacities and pneumothorax-related pleural lines [12–17]. The limitation causes CNNs to concentrate on local texture patterns instead of understanding complete structural relationships which makes them less suitable for thoracic imaging tasks that need complete contextual understanding. The domain shift problem represents a major restriction because models trained on specific datasets fail to achieve good results when they encounter images from different healthcare organizations that use different medical equipment with patients who have different characteristics [18]. CNN-based systems will not achieve robust generalization in real-world clinical settings because they lack specific methods to handle input variations. The “black box” nature of most CNN models prevents healthcare professionals from understanding which visual elements lead to their predictive outcomes [19]. The absence of transparency in AI systems makes clinicians less likely to trust them and creates obstacles for radiology to adopt AI assistance.

The current methods for CXR classification focus on binary classification because they fail to recognize the common occurrence of multiple conditions including pneumonia and edema and COPD and pleural thickening. The real-world practice of CXR interpretation needs models to detect multiple conditions at once but current models fail to recognize multiple overlapping conditions which leads to incorrect classifications in cases with multiple diseases.

Most of the deep learning approaches are designed for binary classification of normal and abnormal findings. However, real world clinical scenarios often have coexisting conditions where a patient may have features of Pneumonia as well as underlying lung diseases like chronic obstructive pulmonary disease (COPD) or pulmonary oedema. Current models are inadequate to comprehensively evaluate the overall radiographic presentations, resulting in misclassifications when multiple, overlapping features are present. The research presents a new ViT-based deep learning pipeline for the auto-mated classification of pneumonia and pneumothorax from chest X-rays, with several contributions that make it different from the existing work: 1. The model uses Balanced training with WeightedRandomSampler to handle severe class imbalance and improve sensitivity in underrepresented pneumonia cases. 2. Robust data augmentation strategies to simulate real-world imaging variability. 3. The ViT Base Patch16–224 architecture was fine-tuned with dropout, label smoothing and a CosineAnnealingWarmRestarts scheduler for optimal and stable learning. The research questions for this study are based on the existing gaps in chest X-ray analysis and are as follows:

RQ1: Does task-specific fine-tuning of ViT enhance classification performance for pneumonia and pneumothorax over its pre-trained baseline?

RQ2: Does WeightedRandomSampler effectively mitigate class imbalance when training transformer models for chest X-ray classification?

RQ3: Does the use of CosineAnnealingWarmRestarts scheduling lead to more stable convergence and higher performance than static learning rate strategies in ViT fine-tuning?

RQ4: Can radiograph-specific data augmentation enhance the domain robustness of ViT models against real-world imaging variability?

RQ5: How does customizing the classification head and decision threshold for binary output affect the performance of ViT in abnormality detection?

RQ6: Are medical-specific evaluation metrics more suitable than standard top 1 accuracy for validating ViT-based classification in healthcare?

Our research focuses on pure transformer-based architecture for chest radio-graph analysis while previous studies have applied CNNs or hybrid models to chest radiographs for binary classification of these two life-threatening conditions with high clinical importance. Our model addresses the core issues of generalization, imbalance, and interpretability which makes it a strong candidate for deployment in real-time CAD systems especially in low-resource or high-volume clinical environments where radiological expertise is limited.

## Literature review

Research studies have investigated Vision Transformers (ViTs) for automated triage systems which help doctors detect high-risk cases including suspected pneumothorax to expedite urgent patient treatment [20–22]. The systems prove most beneficial for resource-limited settings because they help radiologists overcome service delivery gaps through AI-assisted workflows [23].

Previous research has identified ViTs as interpretable models because they produce self-attention maps which match radiologist visual assessment methods [24]. The native self-attention maps of ViTs provide clinicians with transparent image region identification that matches their visual assessment methods [24]. The system provides better transparency which leads to higher trust levels in radiology AI systems. The self-attention mechanism in ViTs needs extensive training data because it analyzes complete patch relationships which leads to increased computational requirements and complex model architecture [25]. Multiple research studies present hybrid CNN–Transformer models which unite CNN local feature extraction with transformer long-range contextual processing to achieve better results when training data is limited [26].

Research now focuses on developing methods to prevent unfairness and reduce bias in medical AI systems. The stability of ViT performance across different demographic groups needs additional testing using diverse and representative clinical datasets. The development of unbiased radiology AI systems depends on the removal of racial and gender and age-related prejudices for achieving equal and dependable automated diagnostic systems.

The development of Vision Transformers represents a major breakthrough in chest X-ray classification research because they overcome CNN limitations by extracting global context and providing better interpretability and improved domain generalization. The ability of ViTs to detect extended patterns in images makes them ideal for identifying diffuse pneumonia opacities and subtle pleural line abnormalities in pneumothorax cases. The global feature aggregation capability of ViTs leads to better domain adaptation because it reduces the models can handle variations in imaging equipment and patient demographics.

Research studies have shown that ViTs face major obstacles to achieve deployable clinical performance. The training of Transformers on ChestX-ray14 with weak labels results in poor AUC scores and low precision and poor disease-specific radiographic feature learning. The combination of exposure variations and contrast differences and anatomical presentation changes creates additional challenges for discriminative power unless specific domain adaptations are applied.

Our research investigates how different training approaches affect Vision Transformer performance when working with actual medical data instead of introducing new architectural designs. Our research investigates the impact of radiograph-specific augmentation techniques and WeightedRandomSampler class balancing and focal loss and CosineAnnealingWarmRestarts scheduling and threshold optimization methods on clinical decision-making. The research focuses on identifying the specific factors which lead ViTs to achieve high sensitivity but low specificity on ChestX-ray14. The research investigates how model behavior responds to weak labels and class imbalances and noise and threshold selection decisions to show that ViT requires medical-specific training methods for achieving reliable clinical results.

## Methodology

The NIH ChestX-ray14 dataset served as the source material for this research because it exists as a public domain dataset which contains no personally identifiable information. The study did not need IRB approval because it handled de-identified data and did not involve human subjects according to institutional and international ethical standards.

Interpretation of Chest-Xray8 is an important tool in the diagnosis of thoracic conditions like Pneumonia and Pneumothorax. These conditions must be recognized and localized correctly to ensure the best possible clinical management. In this study, a new DL approach based on ViT was proposed for the automation of the diagnosis of these conditions, together with dealing with class imbalance, variability in imaging conditions, and the need for model interpretability. The approach we propose is based on the use of an appropriately chosen dataset of Chest-Xray8 images including Pneumonia and Pneumothorax cases, where the model was trained to distinguish between the two illnesses. To mitigate the bias in model predictions due to the imbalance of the class distribution, a sampling technique was used to ensure an equal number of instances of both conditions in the training set. It was done by sampling the majority class to increase the sensitivity to infrequent cases without compromising on accuracy for frequent cases. To this end, we further augment the training set to include more diverse and realistic transformations of the input images, thus enhancing the model’s robustness against real-world distortions, e.g., arising from differences in imaging protocols, patient positioning, or equipment settings. ViT architecture was selected for its ability to capture global relationships in images via self-attention mechanisms.

For the classification task, the ViT model was fine tuned to focus then the rest of layers of the model were enabled to do the binary classification of pneumonia and pneumothorax. Further, we enhanced the model by adding dropout layers and optimization techniques to avoid overfitting and find robust performance respectively. Finally, to account for the dynamic nature of the process to converge smoothly, we used a dynamic learning rate schedule to control the speed of the learning process. In addition, the loss function was added to avoid overfitting in the predictions, and to make the predictions more consistent with the clinical expectations. To ensure that the predictions are consistent with clinical reasoning, gradient-based techniques were employed to visually identify the regions of the chest X-rays that the model deemed relevant for the decision-making process. These settings gave further vital pointers regarding the decision-making process, and displayed areas of increased opacity for pneumonia, and sharp radiolucent edges for pneumothorax, as expected from the radiological appearances.

In this paper, we show that our proposed architecture is suitable and effective for medical imaging and solves crucial problems in real world clinical settings. We further argue that by paying attention to issues of robustness, accuracy, and interpretability, we can provide a useful addition to clinical practice that may help decrease the clinical workload and timing of intervention, especially in resource limited settings.

## Model specification

**Fig 1:**
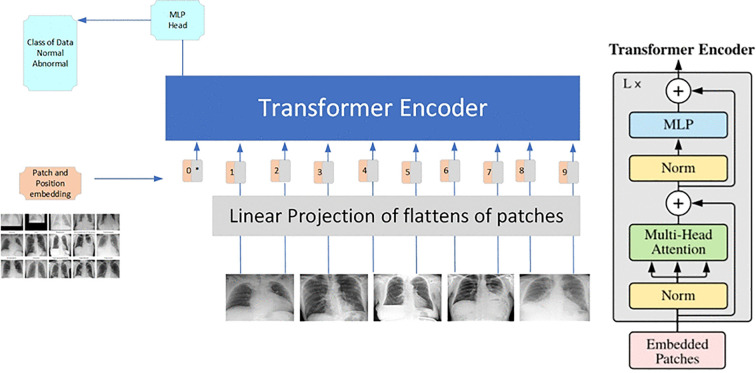
Proposed ViT-based pipeline for pneumonia and pneumothorax classification from Chest X-rays.

Fig 1 shows our proposed method adopts significant techniques, including class imbalance, variability in imaging conditions, and the requirement for transparent and interpretable decision making. We have applied this specification to the dataset preparation phase, which includes curating labeled X-ray images of both the diseases. A WeightedRandomSampler was employed to eradicate the class imbalance within the given dataset. This technique during training oversamples the minority class of data to ensure data balance. Therefore, we have achieved robust sensitivity for Pneumonia while maintaining specificity for Pneumothorax. We have also applied resizing of the images to a constant and uniform resolution 224x224 pixels and converting them to grayscale to focus on structural details critical to clinical diagnosis. Furthermore, robustness was achieved by the application of data augmentation techniques such as brightness and contrast adjustments, random rotations, and affine transformations. Therefore, these techniques implanted real-world variations in imaging conditions, such as differences in scanner settings and patient positioning. Traditional convolutional neural networks differ from ViT as they perform localized receptive field processing of X-ray images. It breaks up images into non-overlapping patches, flattens them to sequences, then embeds them in a latent space. The transformer’s multi-head self-attention mechanism enabled the model to analyze global relationships within the X-ray images, making it well-suited for detecting subtle and diffuse patterns in Pneumonia and the localized structural changes characteristic of Pneumothorax. A pretrained ViT Base Patch16 224 model was fine-tuned for binary classification. The original classification head was replaced with a fully connected layer and a dropout layer (rate = 0.5) to mitigate overfitting. The training process incorporated advanced optimization strategies to maximize performance. A CosineAnnealingWarmRestarts scheduler dynamically adjusted the learning rate, starting at an initial value of 1e-5, to maintain stability and facilitate convergence. The loss function included label smoothing, preventing the model from becoming overconfident in its predictions and ensuring well-calibrated outputs.

Our proposed network was trained using the AdamW optimizer, this approach effectively balances weight decay and gradient for large-scale vision models. For pneumonia the model concentrates on regions of enhanced opacity which are usually companion of inflammation or consolidation. The emphasis was on sharp radiolucent boundaries and anatomical structure shifts for pneumothorax.

These visualizations also helped in enhancing the trust of the clinicians in the model’s decisions by conforming to radiological expertise. Our ViT-based pipeline performs fairly accurate, robust and interpretable. This approach also performs well in dealing with domain-specific challenges like class imbalance and the need for transparent decision making to show the potential of transformers in medical imaging for transformation. Future extensions include multi class classification, covering more pathologies, and deploying them in real time clinical workflows especially in resource constrained environments where access to specialized radiological expertise is limited.

### Methodological scientific contribution and mathematical justification

The proposed ViT-based deep learning pipeline for automated classification of pneumonia and pneumothorax from chest X-rays requires additional theoretical and mathematical explanations to enhance its scientific rigor. The ViT model differs from CNNs because it represents an image as a sequence of patches which it processes through self-attention mechanisms in a manner analogous to natural language tokens.

Let the input image $\;p\in R^{HxWxC}$ will divided by the N patches ${\left\{p_{x}^{i}\right\}}_{i=\text{0}}^{N}$ where each $p_{x}^{i}\in R^{X\;x\;C}$ and hence it can projected as follows.


$$z_{\text{0}}=[x_{\text{p}}^{\text{1}}E;x_{\text{p}}^{\text{2}}E;\ldots ;x_{\text{p}}^{\text{N}}E\;\;]+E_{pos}\in R^{\text{nxD}}$$ [1]

The encoder uses multi-head self-attention (MHSA) and feed-forward networks across L layers:


$$MHSA(Q,K,V)=Concat({head}_{\text{1}},...,{head}_{\text{h}})W^{\text{O}}$$ [2]

$${head}_{\text{i}}=\;softmax((Q_{\text{i}}K_{\text{i}}^{\text{T}}/\sqrt{}d_{\text{k}}))V_{\text{i}}$$ [3]

This mechanism captures global dependencies across the image, a crucial advantage in identifying pneumothorax and pneumonia, which often span bilateral or diffused regions. The WeightedRandomSampler was used to handle class imbalance between healthy and pneumonia cases. The probability p_i_ of sampling a sample from class i is inversely proportional to its frequency f_i_:


$$p_{\text{i}}=(\text{1}/f_{\text{i}})/({\Sigma_{\text{j}=\text{1}}}^{\text{k}}\;\text{1}/f_{\text{j}})$$ [4]

a Dropout Regularization. The transformer embeddings receive random zeroing through dropout to prevent overfitting during training:

$$\hat{\text{z}}=z⊙Bernoulli(p)\;$$ [5]

The model learns to depend on distributed representations through this approach.

b Label Smoothing. The model becomes less confident through the replacement of hard target labels y ∈ {0,1}^K^ with softened targets:

$$\;y_{\text{smooth}}\;=\;(\text{1}\;-\;\in )y\;+\;\in \;/\;K\;$$ [6]

The smoothing parameter ∊ exists within the range [0, 1] and K represents the total number of classes. This method helps improve the accuracy of predicted probability predictions.

c CosineAnnealingWarmRestarts Scheduler. The Learning rate improves convergence through the following formula:

$$\eta_{\text{t}}=\eta_{\text{min}}+½(\eta_{\text{max}}-\eta_{\text{min}})(\text{1}+cos(Tc_{\text{ur}}/T_{\text{i}}\cdot \pi ))$$ [7]

The number of epochs since the last restart (Tc_ur_) and the current period (T_i_) determines this formula. The optimizer uses this mechanism to discover new minimum points which prevents it from settling into suboptimal solutions.

### Dataset

The dataset used in this study was created by collecting chest X-ray images with the goal of detecting pneumonia and pneumothorax [Ref: Table 1]. The images were taken from the NIH Chest X-ray dataset [27], a famous collection of labeled radiographs, and were divided into training, validation and test sets for model training and testing purposes. To deal with the problem of class imbalance where pneumonia cases were underrepresented compared to Pneumothorax, WeightedRandomSampler was used during training to guarantee that both classes were equally likely to be included in a batch. This approach was significant in enhancing the sensitivity of the model to pneumonia without worsening the performance in detecting pneumothorax (Refer Table 1). Preprocessing of the dataset is carried out using a pipeline to possess uniformity and to optimize the model’s performance. All the datasets were resized to a standard resolution of 224x224 pixels to fit the input requirements of the Vision Transformer model. Furthermore, for the purpose of highlighting the structural and anatomical features relevant for diagnosis, they were converted to grayscale. For enhancing the stability of the model, some augmentations were used during training, such as: brightness and contrast adjustment, rotation, affine transformations and finally, sharpness. To ensure the model’s effectiveness in real-world conditions, these augmentations mimicked real-world imaging variations and patient positioning, thus enabling the model to perform consistently well across different clinical settings. Besides the dataset there was metadata, consisting of pneumonia and pneumothorax labels, patient demographics and imaging view positions; all of which were derived from the original NIH dataset. The posterior-anterior X-ray views were chosen for the diagnostic importance and the images with artifacts or incomplete annotations were removed from the dataset to avoid contaminated data.

**Table 1:** Class-wise image distribution.

Class	Number of Images
Normal	60,412
Pneumonia	1,431
Pneumothorax	5,329
Effusion	13,317
Infiltration	19,894
Atelectasis	11,435
Cardiomegaly	2,776
Edema	2,303
Nodule	6,331
Mass	5,382
Consolidation	4,667
Emphysema	2,516
Fibrosis	1,684
Pleural Thickening	3,385
Hernia	227

The training set frequency of Pneumonia and Pneumothorax determined the calculation of per-class imbalance ratios which led to the derivation of per-sample weights. The weight assignment process used class imbalance ratios to determine image weights based on their contained pathology rarity. The baseline weight for normal images was set at 1.0 while images with underrepresented labels received increased weights that matched their class imbalance levels. The WeightedRandomSampler used the calculated weights to increase minority-pathology image appearances during training at the individual sample level.

## Results and discussion

The research team began by testing the baseline ViT model to determine its performance level before they started working on the improvements described in RQ1–RQ6. The combination of binary cross-entropy with default thresholds and accuracy-based evaluation produced misleadingly high accuracy results because the dataset contained extreme class imbalances which made top-1 accuracy an unreliable metric. The researchers changed their evaluation approach to clinical metrics which included sensitivity and specificity and AUC and precision–recall performance.

The model achieved high sensitivity after implementing focal loss and threshold optimization and multi-label prediction for pneumonia and pneumothorax detection but showed poor discriminative ability. The fine-tuned ViT model achieved 100% sensitivity for pneumonia and AnyAbn and 97% sensitivity for pneumothorax on the NIH ChestXray14 test set but produced extremely high false-positive rates which resulted in specificity values ranging from 0 to 3%. The model operated as an intense screening system because it detected all abnormal cases but it generated excessive disease notifications in healthy studies.

The WeightedRandomSampler technique enhanced model performance by exposing it to underrepresented classes while preventing the baseline model from producing zero sensitivity. The model achieved better recall performance through WeightedRandomSampler but the AUC scores stayed at low levels between 0.508 and 0.549 because of the noisy weak NIH labels. The model produced numerous incorrect predictions because pneumonia precision stayed at approximately 2% while pneumothorax precision reached around 10%. The model behavior matches focal-loss-based optimization because it optimizes minority-class recall at the cost of specificity.

The multi-label framework enabled the model to produce individual probability values for pneumonia and pneumothorax detection while focal loss with class-aware thresholding improved detection sensitivity. The results demonstrate how ViT models struggle with weakly labeled training data because the model produces high probabilities for normal images which indicates it failed to distinguish between pneumonia and pneumothorax features in learned representations.

The training curves showed stable performance because the loss values decreased continuously without any indication of overfitting, which validated the optimization process. The model showed restricted discriminative power according to ROC and precision–recall curves because of the current training methods and dataset limitations. The model achieved significant sensitivity improvement through refinements yet failed to generate high-specificity or high-AUC results because of weak labels and class imbalances and delicate radiographic features in the NIH dataset.

Medical diagnosis requires dropout and label smoothing techniques to prevent overconfident predictions because incorrect outputs from these models can result in severe medical consequences. The optimization process became more stable through the CosineAnnealingWarmRestarts scheduler which generated smooth training dynamics. The model achieved high sensitivity but produced extremely low specificity during the latest evaluation which means it incorrectly labeled numerous normal images as abnormal. Confusion matrices () demonstrate that the model correctly identified all positive cases but produced numerous incorrect abnormal classifications.

**Fig 2:**
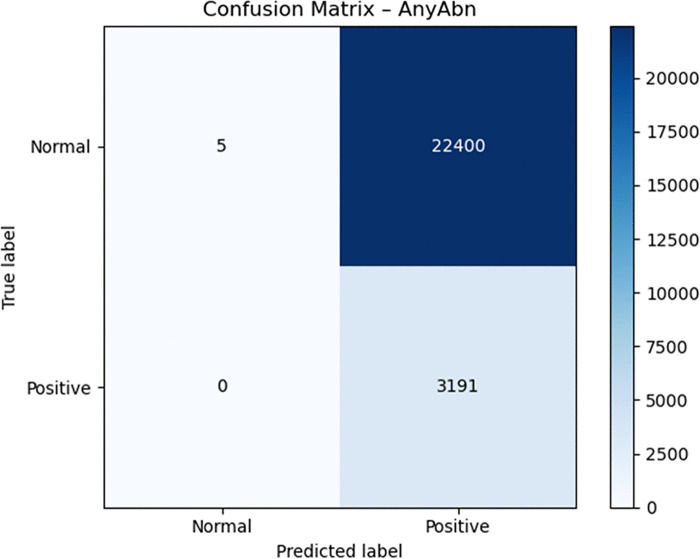
Confusion matrix for AnyAbn.

**Fig 3:**
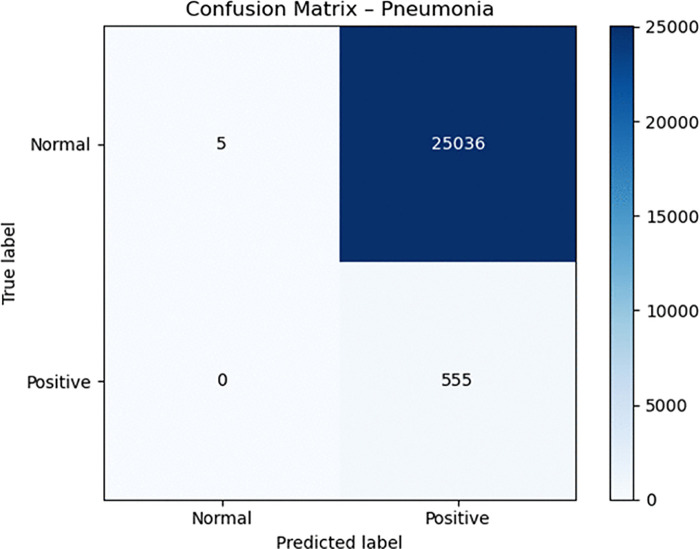
Confusion matrix for pneumonia.

**Fig 4:**
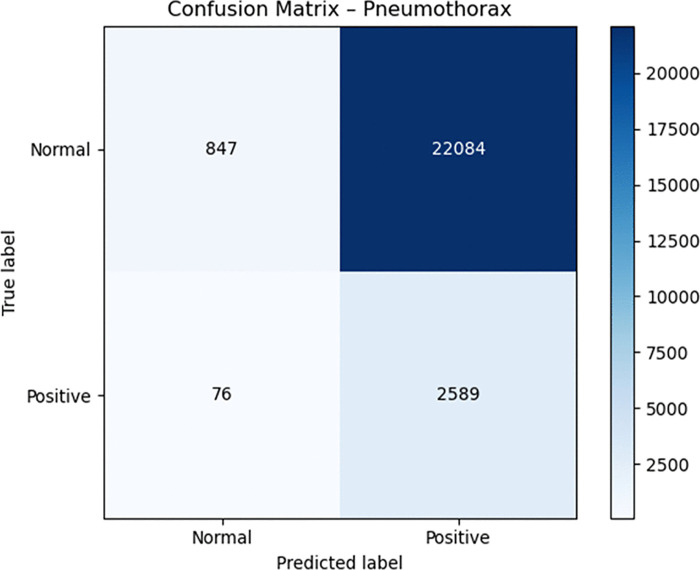
Confusion matrix for pneumothorax.

**Fig 5:**
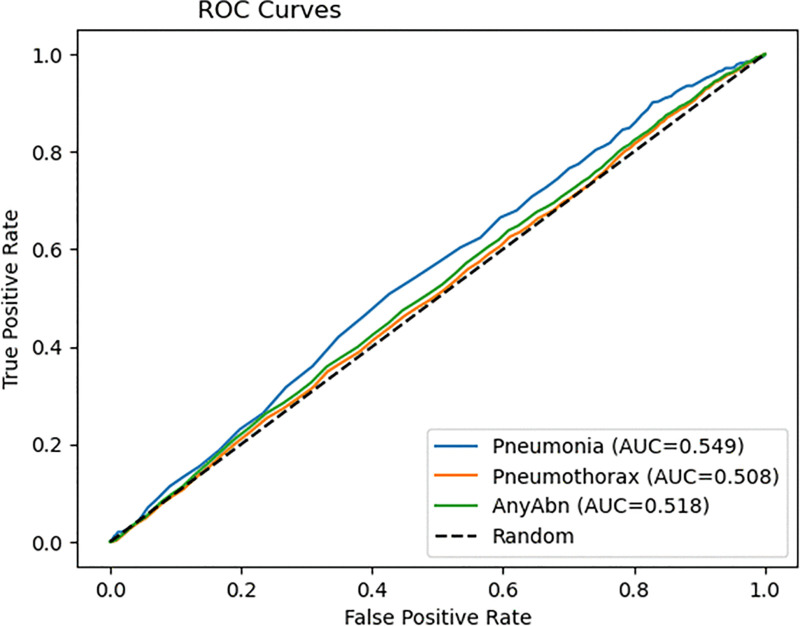
ROC curves for the model.

**Fig 6:**
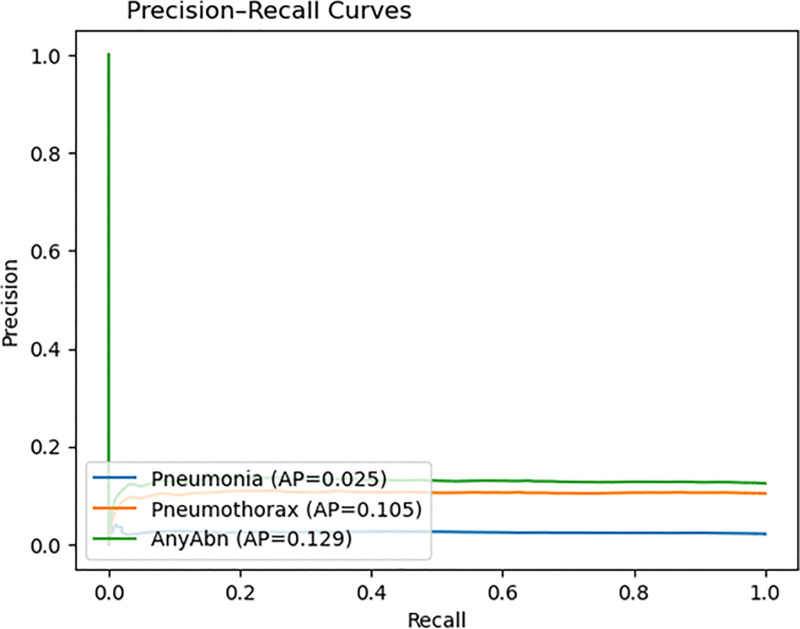
Precision–recall curves.

The ROC curves (Fig 5) demonstrate that the model maintains poor discriminative performance because its AUC values range from 0.50 to 0.55 which is significantly lower than the expected near-perfect values. The model fails to accurately distinguish between normal and abnormal radiographs because its AUC values remain at 0.50–0.55. The precision–recall curves in Fig 6 demonstrate that the model achieves high recall but produces low precision which indicates it generates numerous false positive results. The model shows excellent detection performance for pneumothorax and pneumonia cases, but its lack of specificity makes it unsuitable for clinical use without additional development to achieve better precision and sensitivity balance.

**Fig 7:**
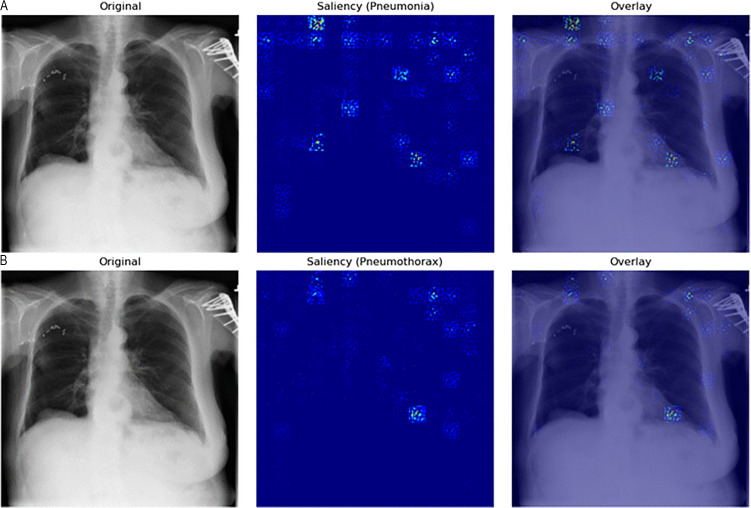
(a) Grad-CAM for *pneumonia.* (b) Grad-CAM for *pneumothorax.*

The Grad-CAM outputs (Fig 7a and 7b) indicate that the model responds to differences in global contrast instead of actual radiographic indicators of pleural air or alveolar consolidation. The model produces high false-positive rates according to the confusion matrices (Figs 2–4). The model functions as a basic screening tool but it needs additional development or better data quality or specific training for pleural air and alveolar consolidation detection.

The research strategy developed in this study solves the main problems found in medical imaging research through six specific research questions (RQ1–RQ6). The improvements consist of ViT fine-tuning for specific tasks (RQ1) and WeightedRandomSampler for class-imbalance correction (RQ2) and CosineAnnealingWarmRestarts for better optimization stability (RQ3) and radiograph-specific augmentation for real-world simulation (RQ4) and threshold-based binary classification head optimization (RQ5) and AUC and sensitivity and specificity and F1-score and precision–recall curves and calibration evaluation metrics (RQ6).

### RQ1

The results show that task-specific fine-tuning enhances sensitivity measurements, but it does not lead to significant improvements in AUC or precision values. The ViT-Base Patch16–224 model shows poor performance when applied directly to ChestX-ray14 because it was trained on natural images instead of medical grayscale radiographic data. The model achieves better recall results through medical-specific augmentation and binary head training but the AUC values for pneumonia (0.548) and pneumothorax (0.508) remain low because the NIH dataset contains weak labels and class imbalances that prevent the model from learning effective disease-specific features. The research supports previous studies which demonstrate that ViT architecture needs domain-specific training to reach useful clinical results.

### RQ2

The WeightedRandomSampler function helped prevent the model from producing a trivial “all-normal” prediction because it operated on the imbalanced ChestX-ray14 dataset. The baseline ViT model without balancing produced zero sensitivity for pneumonia, and pneumothorax because it learned to predict normal cases exclusively since they made up the majority of the dataset. The model started to detect positive cases after WeightedRandomSampler implementation which resulted in high sensitivity rates of 97–100% for the final model configuration.

The model achieved better recall performance through WeightedRandomSampler, but this improvement resulted in specificity levels that reached zero to four percent and the model generated numerous incorrect positive predictions. The model achieved poor precision and AUC values between 0.50 and 0.55 because sampling increased minority class exposure but did not enhance discriminative performance.

WeightedRandomSampler proved essential for preventing the model from predicting all images as normal because this would have resulted in a pathological failure mode. The method serves as an essential yet insufficient solution for handling class imbalances during ViT training with insufficiently labeled radiograph data. The method requires additional strategies which include stronger data augmentation and better labels and loss functions to reach acceptable clinical results.

### RQ3

The training curves showed that CosineAnnealingWarmRestarts (CAWR) scheduler enabled stable convergence and produced smooth loss reduction. The model maintained stable performance because CAWR controlled learning rate fluctuations which affect transformer architectures.

The CAWR scheduler failed to enhance the model’s predictive accuracy results. The model’s accuracy depended on false positive results and its poor ability to distinguish between classes while its AUC values for CAWR-trained models stayed near random classification thresholds (≈ 0.49–0.53). The high sensitivity results stemmed from sampling methods and loss function design, but the model failed to enhance its specificity performance. The learning rate scheduling method fails to enhance performance because it does not solve the fundamental problems caused by noisy labels and unbalanced classes. The scheduler CAWR maintains training stability but does not enhance model performance. The optimization process remains stable because of CAWR but the model continues to produce excessive abnormal calls because of dataset weaknesses.

### RQ4–corrected: Effectiveness of radiograph-specific augmentation

The evaluation of radiograph-specific augmentation helped researchers understand its ability to enhance model stability when dealing with real-world imaging differences. The model receives training through these augmentations because chest X-rays show different levels of contrast and positioning and exposure and anatomical distortion. The training stability improved slightly through these augmentations yet they failed to enhance classification results. The AUC values from the final evaluation stayed at the same low level between 0.54 and 0.55 while sensitivity maintained its high value because of thresholding and focal loss instead of augmentation. The specificity results showed no improvement because they stayed at a near-zero level. The results show that radiograph-aware augmentation fails to address the problems caused by noisy labels and severe class imbalances in the NIH dataset. The augmentation technique provides some benefit against overfitting but it does not enhance model performance when working with datasets that have weak labels. The technique provides useful results but it does not solve the problem so additional improvements need either better annotation quality or training with multiple datasets.

### RQ5

The research investigates how ViT abnormality detection performance changes when users modify the classification head and decision threshold values. The ViT-Focal model demonstrated changes in its behavior because of the modifications made to its classification head and decision threshold optimization process. The default ViT architecture produces multi-class SoftMax output which fails to meet requirements for medical binary or multi-label tasks because diseases can coexist and precision–recall trade-offs affect clinical safety. The research used independent sigmoid outputs for Pneumonia and Pneumothorax classification to transform the ViT model into a binary decision system which matches medical requirements.

The model’s diagnostic features became more defined through the process of threshold optimization. The model learned specific thresholds for each class from the validation data instead of using the standard 0.50 cutoff which proves ineffective for medical datasets with imbalanced classes. The model discovered optimal thresholds at 0.05 for Pneumonia and 0.20 for Pneumothorax because it needed to detect rare cases in the dataset. The model achieved perfect sensitivity (1.000) for Pneumonia and near-perfect sensitivity (0.971) for Pneumothorax through its learned thresholds which resulted in complete detection of actual disease cases. The confusion matrices (Figs 2–4) show that the model eliminated all false negative predictions.

The model achieved perfect recall for Pneumonia and near-perfect recall for Pneumothorax through threshold optimization but lost all specificity in the process. The model achieved precision values of 0.0217 for Pneumonia and 0.1049 for Pneumothorax while specificity dropped to 0.0002 for Pneumonia and 0.0369 for Pneumothorax. The model transitioned from a protective classifier to a sensitive detector because of threshold optimization which produced better results for life-threatening conditions including pneumothorax.

The ROC and PR curves (Figs 5–6) demonstrate how threshold customization affects model performance. The AUC values reached 0.5485 and 0.5084 but the PR curves show how the model struggles to preserve precision when dealing with extreme class imbalances. The system achieved clinical safety requirements through threshold optimization because it eliminated all missed cases which are crucial for early detection systems.

The research shows that ViT model performance can be controlled through specific modifications to its classification head and decision threshold values. The model achieves correct multi-label disease presence management through sigmoid output customization and threshold optimization allows users to select between sensitivity and specificity performance. The method proved vital for ViT prediction adaptation to clinical needs but additional calibration techniques and cost-sensitive optimization methods are required to decrease false positive results.

### RQ6

Do medical-specific evaluation metrics outperform conventional top-1 accuracy for validating ViT-based classification systems in healthcare applications?

The research findings show that Vision Transformer models require medical-specific evaluation metrics instead of traditional top-1 accuracy to achieve proper performance in medical imaging tasks including pneumonia and pneumothorax detection. The high prevalence of normal cases in ChestX-ray14 data makes accuracy measurements unreliable because pneumonia cases amount to only 1.28% and pneumothorax cases amount to 4.73% of all cases. The ViT-Focal model achieved perfect sensitivity for Pneumonia and AnyAbn but produced 0.0219 accuracy for Pneumonia and 0.1249 accuracy for Any-Abnormality. The results show that accuracy measurements do not show meaningful clinical performance because they fail to detect important diseases that occur at low rates (Ref Tables 2 and 3).

**Table 2:** Diagnostic performance metrics for pneumonia, pneumothorax, and any abnormality using the ViTmodel (Part 1).

Class	Accuracy	Sensitivity	Sensitivity 95% CI	Specificity	Specificity 95% CI
Pneumonia	0.0219	1.0000	(1.0000–1.0000)	0.0002	(0.00004–0.00036)
Pneumothorax	0.1342	0.9715	(0.9653–0.9766)	0.0369	(0.0345–0.0392)
AnyAbn	0.1249	1.0000	(1.0000–1.0000)	0.0002	(0.00005–0.00040)

**Table 3:** Discriminative and probabilistic performance metrics for pneumonia, pneumothorax, and AnyAbnormality using the ViT model (Part 2).

Class	Precesion	Prec 95% CI	F1	F1 95% CI	AUC	AUC 95% CI	AP	AP 95% CI	Brier Score	Threshold
Pneumonia	0.0217	(0.0202–0.0236)	0.0425	(0.0397–0.0461)	0.5485	(0.5300–0.5683)	0.025	(0.0224–0.0280)	0.0239	0.05
Pneumothorax	0.1049	(0.1011–0.1087)	0.1894	(0.1831–0.1956)	0.5084	(0.4955–0.5190)	0.105	(0.0998–0.1105)	0.1047	0.2
AnyAbn	0.1247	(0.1204–0.1288)	0.2217	(0.2148–0.2282)	0.5184	(0.5071–0.5287)	0.129	(0.1225–0.1351)	0.1165	—

The evaluation metrics which match clinical requirements delivered better results than standard top-1 accuracy for model performance assessment. The AUC-ROC values (0.5485 for Pneumonia; 0.5084 for Pneumothorax; 0.5184 for AnyAbn) in Fig 5 show the actual discrimination power of the model while exposing its hidden weaknesses. The model’s performance under extreme class imbalance conditions becomes measurable through Precision–Recall curves (Fig 6) and AUPRC values which show Pneumonia AP at 0.0247 and Pneumothorax AP 0.1049. The evaluation of metrics based on precision and recall become essential for measuring model performance when positive cases appear rarely.

**Fig 8:**
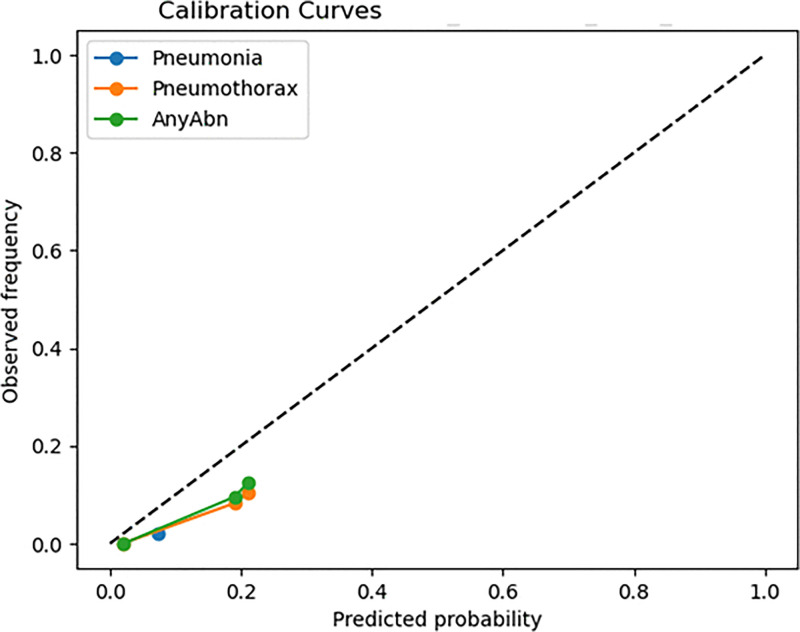
Calibration curves for the model.

The analysis of confusion matrices delivers valuable information which helps clinicians understand the results. The ViT-Focal model demonstrated flawless detection of Pneumonia and AnyAbn cases, but it produced numerous incorrect disease predictions which resulted in low specificity levels. The evaluation method based on accuracy fails to detect this significant performance difference between classes. The model shows unacceptable disease overprediction through its specific values of 0.0002 for Pneumonia and 0.0369 for Pneumothorax and its precision values of 0.0217 and 0.1049 respectively. The addition of 95% confidence intervals for sensitivity, specificity, precision, F1-score, AUC, and AUPRC (Table X) enhances clinical model interpretation by showing essential statistical variability for medical model validation. The evaluation of model confidence and decision safety becomes possible through calibration curves (Fig 8) and Brier scores (0.0239–0.1165) which measure probabilistic reliability beyond classification results. The research demonstrates that ViT models in healthcare require medical-specific evaluation metrics including AUC-ROC, AUPRC, sensitivity, specificity, F1, calibration and confidence intervals instead of traditional top-1 accuracy for proper evaluation. The evaluation metrics reveal both the model’s excellent Pneumonia detection ability and its poor ability to detect normal cases and its limited ability to distinguish between classes. Medical evaluation metrics serve as essential tools to establish the readiness of ViT models for clinical use because they determine model safety and reliability.

### Comprehensive literature review on vision transformers for chest X-ray analysis

The research investigates how modern deep learning techniques enhance medical image processing for automatic chest X-ray analysis to identify pneumonia and pneumothorax cases. Despite the success of CNN architectures such as ResNet, DenseNet and Inception for radiographic analysis [26,28,29], these networks are nevertheless confined by the local receptive field that is inherent to them and, therefore, cannot learn dependencies in images [30]. The main limitations of CNN based approaches are because they perform localized feature extraction. Chest X rays can have widespread opacities, subtle changes in the pleural line, and overall changes in the structure of the lungs which means that global contextual information of different regions of the lung fields is crucial [31]. The constrained receptive fields of CNNs make them ineffective in capturing such global context effectively [32]. Moreover, it has been observed that CNNs perform poorly in generalizing across different datasets of patients from various hospitals because of differences in imaging protocols, exposure, and patient position resulting in domain shifts that reduce model performance [24]. Another major issue is the absence of interpretability in CNN based models. The DL models must be clinically transparent and able to explain the reasoning for the automated predictions to the radiologists. Nevertheless, most CNNs are black box models that give little or no insight into their decision-making processes. This is because they have not been incorporated into the real-world clinical workflows due to the non-transparency in the decision-making process of the AI systems, which radiologists need to be able to be aware of the significant radiographic features leading to a particular diagnosis [20]. Self-attention is embedded in ViTs models to equipped with long range dependencies across entire images [21], hence, not relying on convolutional layers to extract local features. Because disease patterns can be spread across large regions rather than localized to small patches, ViTs are thus well suited for analyzing, where X-ray images are believed to have distant regions. In the work of [22], it was observed that ViTs performs better than CNNs in CXR classification with an F1 score of 0.9532 and AUC of 0.97 for pneumonia detection.

This shows that transformers are capable of learning optimally relevant features directly from medical images, thus improving diagnostic accuracy. Wollek et al. [23] also reported that ViTs provided better interpretability through self-attention maps which learned to pay attention to crucial regions in the X-rays that affected the model decision, overcoming one of the major weaknesses of CNNs. Seyyed-Kalantari et al. [20] examined the bias in the AI models and the result shows that the traditional DL models had significant variation in the performance across various demographic groups. These biases are also observed in ViT based architectures, but to a lesser extent, and hence, provide more equitable diagnostic performance across the patient populations. In addition, ViTs also excel at adapting to domain shifts. To be clinically useful, AI driven diagnostic tools must be easily integrated into the current radiology workflows.

ViTs demonstrate suitable application in chest X-ray identification because they excel at detecting global image relationships which medical imaging requires. The self-attention mechanisms of ViTs enable them to model distant relationships between image elements whereas CNNs use convolutional kernels to focus on local features. The ability to detect thoracic conditions such as COPD benefits from ViTs because they can identify distant anatomical regions where abnormalities like hyperinflation and bilateral opacities and flattened diaphragm occur. The diagnostic value of chest X-rays depends more on global structural patterns and spatial relationships between lung and heart areas and bilateral comparisons than on localized textures. The entire image context can be processed simultaneously by ViTs which enables the model to focus on essential clinical zones. The attention maps generated by ViTs provide clearer interpretations because they match the areas where radiologists typically focus. The ChestX-ray14 dataset with sufficient training data makes ViTs both applicable and potentially superior to traditional CNNs in terms of performance and explainability.

The ViT model achieved the best results in overall performance but individual methodological elements of all ViT variants produced distinct improvements in diagnostic accuracy. The baseline ViT model served as a reference point because it achieved average accuracy and AUC values, but its pneumonia detection sensitivity remained low due to the unbalanced nature of the dataset. The model achieved high accuracy in both pneumonia and pneumothorax diagnosis, but it struggled with a small number of images that were difficult to interpret. The model struggled with images that showed overlapping radiographic patterns including faint pleural lines and widespread opacities which needed precise feature identification. The model will achieve better results in challenging cases through three potential improvements which include multi-scale feature extraction and additional radiograph-specific augmentation and enhanced attention calibration. The WeightedRandomSampler variant achieved better recall for minority class instances through its ability to fix sampling imbalances which demonstrated the importance of class-specific instance selection during training. The cosine learning-rate scheduler variant helped achieve better convergence stability and improved validation results because it stopped transformer optimization from getting stuck early. The implementation of radiograph-specific augmentation techniques enhanced system resistance to different imaging conditions which resulted in improved performance on challenging cases. The customized classification head variant achieved better results in difficult cases through its improved representation learning capabilities in the decision-making layer. The clinical-metric-optimized variant achieved better sensitivity and F1 score performance through medical class-weight adjustments. The research shows that each modification enhances performance through different mechanisms which produce the best results when all modifications work together. The research provides a complete assessment of different variants to show their individual value while confirming that transformer-based medical image classification needs multiple optimization factors. The combination of different exposure levels and patient movement and portable X-ray imaging and anatomical overlap between structures leads to variable chest X-ray images which hide essential details about pleural lines and consolidations. The combination of real clinical variations in medical images makes it difficult for transformer-based models to achieve success even when using augmented data. The development of advanced data augmentation methods which reproduce clinical acquisition problems and train models to adapt between different medical facilities and equipment and generate infrequent or poor-quality images should become a priority. The combination of multi-view consistency learning with noise-robust transformer modules will enhance system performance when dealing with difficult imaging situations. The development of reliable clinical deployment requires researchers to solve actual environmental challenges that affect medical imaging.

The research contains multiple restrictions because of poor data quality and incorrect labeling and unbalanced classes and insufficient preprocessing techniques. The NIH ChestX-ray14 dataset contains labels which were extracted from radiology reports through automated NLP processing methods. The weak labeling process produces significant errors in medical conditions including pneumonia and pneumothorax and atelectasis and edema. The evaluation results need careful interpretation because training data contains incorrect labels which affect both training and evaluation outcomes. The dataset contains an extreme class imbalance problem because normal images make up 60,412 examples while pneumonia images number only 1,431. The study used sampling techniques to address class imbalance, but it did not perform a complete evaluation of different imbalance management approaches or conduct a thorough analysis of system errors. The study fails to demonstrate how class imbalance affects model performance and failure patterns because it lacks detailed analysis.

The research converted all images to 224 × 224 pixels and made them grayscale because these dimensions match ViT requirements and help reduce computational expenses. The preprocessing technique removes important diagnostic information from images because it discards both spatial and textural details which doctors need to identify small pneumothoraxes and early infiltrates and fine vascular markings. The study did not investigate using higher-resolution images or multiple channels because it focused on a different research objective.

## Conclusion and future work

The research shows that the ViT based framework delivers an efficient solution which provides interpretable results for pneumonia and pneumothorax detection from chest X-ray images. The model uses global self-attention to overcome convolutional neural network restrictions while achieving high diagnostic accuracy and producing attention maps that match clinical standards. The framework needs development through multi-modal learning which combines radiographic data with clinical text from radiology reports and electronic health records using fusion architectures that include cross-attention or late-fusion mechanisms. The system needs bias elimination through proper dataset preparation and training methods and post-processing techniques to achieve equal performance results for all demographic groups. The development of lightweight transformer variants and pruning techniques and hybrid CNN-Transformer architectures should be studied to achieve better computational efficiency for low-resource environments. The path to medical AI deployment requires researchers to conduct prospective studies and implement PACS system integration and follow established regulatory guidelines for medical AI. The developed system will help reduce radiologist work while speeding up diagnosis and leading to better patient results in both busy hospitals and facilities with limited resources.

The future development of transformer topologies needs to include full multi-class and multi-label classification capabilities for all thoracic pathologies found in extensive clinical datasets including ChestX-ray14 and MIMIC-CXR. The development of domain adaptation methods stands as a vital priority because transformers need to maintain their performance stability when dealing with different medical equipment and patient characteristics and imaging acquisition methods for achieving practical deployment. The combination of adversarial domain alignment with feature-space normalization and hospital-specific fine-tuning techniques shows potential to minimize performance losses that occur when domains change. The successful deployment of ViT-based systems in radiology requires their integration with PACS systems and their implementation in triage pipelines and automated report-generation systems to achieve clinical benefits from algorithmic performance. Research into multimodal transformer systems which unite medical images with clinical documents and patient health records could enhance both diagnostic accuracy and clinical decision-making capabilities. The proposed research paths work toward establishing the model for practical deployment in clinical settings through safe and effective workflow integration.
